# *TERT* Promoter Mutation as an Independent Prognostic Marker for Poor Prognosis MAPK Inhibitors-Treated Melanoma

**DOI:** 10.3390/cancers12082224

**Published:** 2020-08-09

**Authors:** Pauline Blateau, Etienne Coyaud, Estelle Laurent, Benoit Béganton, Vincent Ducros, Géraldine Chauchard, Julie A. Vendrell, Jérôme Solassol

**Affiliations:** 1 Laboratoire de Biologie des Tumeurs Solides, Département de Pathologie et Oncobiologie, Centre Hospitalier Universitaire de Montpellier, 34000 Montpellier, France; p-blateau@chu-montpellier.fr (P.B.); b-beganton@chu-montpellier.fr (B.B.); v-ducros@chu-montpellier.fr (V.D.); g-chauchard@chu-montpellier.fr (G.C.); j-vendrell@chu-montpellier.fr (J.A.V.); 2 Institut de Recherche en Cancérologie de Montpellier, INSERM, Université de Montpellier, Institut du Cancer de Montpellier, Université de Montpellier, 34000 Montpellier, France; 3 Laboratoire Protéomique Réponse Inflammatoire Spectrométrie de Masse (PRISM), INSERM U1192, Université de Lille, Centre Hospitalier Universitaire Lille, F-59000 Lille, France; coyaud@gmail.com (E.C.); estellelaurent81@gmail.com (E.L.)

**Keywords:** *BRAF*^V600^, melanoma, co-occurring mutation, *TERT* promoter, prognosis factor, targeted therapies

## Abstract

Although the development of mitogen-activated protein kinase (MAPK) inhibitors has greatly improved the prognosis of *BRAF*^V600^ cutaneous melanomas, the identification of molecular indicators for mutated patients at risk of early progression remains a major issue. Using an amplicon-based next-generation-sequencing (NGS) assay that targets cancer-related genes, we investigated co-occurring alterations in 89 melanoma samples. We analyzed both their association with clinicopathological variables and clinical significance in terms of progression-free survival (PFS) and overall survival (OS) according to *BRAF* genotyping. Among co-occurring mutations, *TERT* promoter was the most frequently mutated gene. Although no significant difference in PFS was observed in the presence or absence of co-occurring alterations to *BRAF*^V600^, there was a trend of longer PFS for patients harboring *TERT* c.-124C>T mutation. Of most interest, this mutation is an independent marker of good prognosis in subgroups of patients with poor prognosis (presence of brain metastasis and elevated level of lactate dehydrogenase, LDH). Moreover, combination of elevated LDH level, presence of brain metastasis, and *TERT* c.-124C>T mutation was identified as the best fit model for predicting clinical outcome. Our work revealed the potential interest of c.-124C>T status determination in order to refine the prognosis of *BRAF*^V600^ melanoma under mitogen-activated protein kinase (MAPK) inhibitors.

## 1. Introduction

Over the past few years, the molecular characterization of melanomas has greatly improved, with an emphasis on alteration of cell signaling pathways [1,2]. Approximately 40% of patients with melanoma exhibit exon 15 *BRAF* mutations in cancer cells, resulting in constitutive activation of the mitogen-activated protein kinase (MAPK) cascade. A therapeutic strategy based on dual inhibition of the MAPK pathway through targeting BRAF and MEK proteins with BRAF inhibitors (e.g., dabrafenib or vemurafenib) in combination with MEK inhibitors (e.g., trametinib or cobimetinib) has significantly improved progression-free survival (PFS) and overall survival (OS) in melanoma patients harboring activating *BRAF* mutations [3]. Concurrently, immune checkpoint inhibitors targeting Programmed Death -1 (PD-1) and cytotoxic T-lymphocyte associated protein 4 (CTLA-4) showed clinically significant improvements in OS in molecularly unselected populations of advanced melanoma patients. Recent data support the hypothesis that these therapies also provide clinical benefit in melanoma patients with activating *BRAF* mutations [4].

Although these therapies have significantly improved the prognosis of melanoma advanced forms, their effectiveness in practice remains subject to significant interpersonal variation between patients, with some patients showing primary resistance or early progression. Within this group, prognostic factors conventionally useful in distinguishing individuals at risk of poor clinical outcome or progression from others include the following: stage of disease; baseline serum lactate dehydrogenase (LDH) levels; presence of brain metastases, and the Eastern Cooperative Oncology Group’s (ECOG PS) baseline performance status [5]. However, these prognostic features have been validated years before the advent of targeted therapies and use of BRAF and MEK inhibitors. Hence, they appear as poorly suitable for the genotyping status-based stratification of melanoma patients.

With the recent emergence of next-generation-sequencing (NGS) analyses, concomitant somatic genomic alterations have been identified in samples of *BRAF* mutant melanomas [6,7,8,9], such as *CDKN2A*, *PTEN*, *RAC1,* and *TERT* promoter [10]. Most of these co-occurring mutations have been studied individually, leading in some cases to the identification of resistance mechanisms against BRAF and MEK inhibition therapy, such as the activation of the MAPK or PI3K/AKT pathway [11,12,13,14]. However, the relationships between *BRAF*^V600^ mutation, concomitant molecular alterations, and clinicopathological parameters as prognosis markers have been barely investigated in melanoma.

Thus, there is a critical need for identifying and validating new tissue biomarkers able to refine patient prognosis and classify them into specific subgroups more or less likely to progress early under targeted therapy, especially in patients with distant metastasis. To tackle this important clinical question, we performed NGS analysis on a cohort of 113 cutaneous melanoma patients focusing on 35 clinically relevant cancer hotspot regions. We analyzed the frequency and type of co-occurring mutations and their association with *BRAF* status. We also evaluated the correlation between concomitant genomic alterations in *BRAF* mutant melanomas with their clinical and pathological characteristics, as well as their potential synergistic effect on patient outcome.

## 2. Results

### 2.1. Patient Characteristics

**Figure 1:**
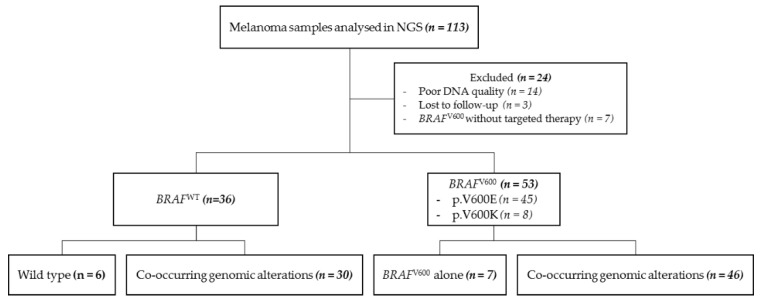
Analytical flowchart of the study. *BRAF*^WT^: *BRAF* wild type, NGS: next generation sequencing.

A total of 113 samples of cutaneous melanoma were collected and exhaustively analyzed by NGS between April 2014 and September 2019 at the Pathology Laboratory of the University Hospital of Montpellier, France, to assess the presence of molecular alterations (Figure 1). Patients eligible for this retrospective study were diagnosed either for primary or recurrent metastatic melanoma. Their clinicopathological features are shown in Table S1.

The dropout (*n* = 24) was based on poor DNA quality or lost-to-follow-up. Specifically, we observed that 53 samples (59.6%) harbored a *BRAF*^V600^ mutation and 36 samples (40.4%) were *BRAF* wild type (*BRAF*^WT^) (Figure 1).

### 2.2. NGS Analysis and Co-Occurring Genetic Alteration Detection

We next determined the prevalence of co-occurring aberrations in the 89 formalin-fixed paraffin-embedded tumor samples analyzed by NGS.

**Figure 2:**
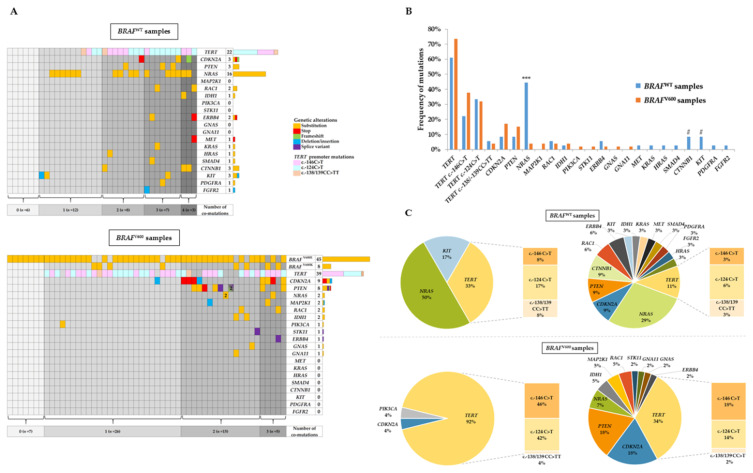
Mutational landscape profiling in *BRAF*^WT^ and *BRAF*^V600^ samples. (**A**) Co-occurring genetic alterations detected in *BRAF*^WT^ (*n* = 36, upper panel) and *BRAF*^V600^ samples (*n* = 53, lower panel). Alteration types are specified (substitution, stop, frameshift, deletion/insertion, or splice variant), except for *TERT* c.-146C>T, c.-124C>T, or c.-138/139CC>TT mutations. The total number of mutations is shown for each mutated gene in the histogram at the right side of the figure. (**B**) Frequency of mutated genes in *BRAF*^WT^ and *BRAF*^V600^ samples. *** *p* < 0.001; ^#^ *p*-values close to significance (*p* = 0.06). (**C**) Percentage of mutated genes in *BRAF*^WT^ (upper panel) and *BRAF*^V600^ (lower panel) samples according to the number of co-occurring genetic alterations. Left pie charts show the percentage of mutated genes in samples harboring a single genetic alteration in *BRAF*^WT^ (*n* = 12) and *BRAF*^V600^ (*n* = 26). Right pie charts show the percentage of mutated genes in samples harboring several genetic alterations in *BRAF*^WT^ (*n* = 18) and *BRAF*^V600^ (*n* = 20).

In *BRAF*^WT^ specimens, 30 samples (83.3%) analyzed through our panel NGS assay revealed genetic alterations other than *BRAF*^V600^ (Figure 2A, upper panel). A single mutation was found in 12 samples (33.3%), whereas multiple co-occurring mutations were found in 18 patients (50.0%), with two, three, and four mutations in eight (22.2%), seven (19.4%), and three (8.3%) specimens, respectively. In descending order of frequency, the most common mutations were found in *TERT* promoter (*n* = 22, 61.1%), then in *NRAS* (*n* = 16, 44.4%), *KIT*, *CDKN2A*, and *CTNNB1* genes (*n* = 3 for each, 8.3%) (Figure 2A,B). The predominant *TERT* promoter mutation in *BRAF*^WT^ samples was c.-124C>T, detected in 12 (33.3%) cases, whereas c.-146C>T and c.-138/139CC>TT mutations were present in eight (22.2%) and two (5.6%) cases, respectively. Several other mutations were detected at a lower incidence with two mutations in *RAC1*; two in *ERBB4*; and one in *MET*, *KRAS*, *HRAS*, *SMAD4*, *PDGFRA*, and *FGFR2.*

Among *BRAF*^V600^ specimens, 7 samples (13.2%) exhibited a unique *BRAF*^V600^ mutation, while 46 samples (86.8%) had at least one co-occurring mutation (Figure 2A, lower panel). Twenty-six samples (49%) had a single additional mutation to *BRAF*^V600^, whereas extra-aberrations on other genes were present in 20 samples (37.7%), with two and three mutations for 15 samples (28.3%) and 5 samples (9.4%), respectively. The prevalence of co-occurring mutations is shown in Figure 2B. *TERT* promoter was the most frequent genetic alteration in *BRAF*^V600^ samples (*n* = 39, 73.6%) with a predominance of c.-146C>T genotype (*n* = 20, 73.6%) compared with c.-124C>T genotype (*n* = 17, 32.1%). *CDKN2A* and *PTEN* were commonly mutated, but at a lower frequency, in nine (17.0%) and eight (15.1%) cases, respectively. The remaining co-occurring mutations were detected in *NRAS* (*n* = 2), *MAP2K1* (*n* = 2), *RAC1* (*n* = 2), *IDH1* (*n* = 2), *PIK3CA* (*n* = 1), *STK11* (*n* = 1), *ERBB4* (*n* = 1), *GNAS* (*n* = 1), and *GNA11* (*n* = 1).

When we compared the frequencies of co-occurring mutations variations between the *BRAF*^V600^ and *BRAF*^WT^ patients, we only found a statistically significant difference between these two groups for *NRAS* (3.8% in *BRAF*^V600^ samples versus 44.4% in *BRAF*^WT^ samples, *p* < 0.001) (Figure 2B). Of note, some mutations were exclusively detected in *BRAF*^WT^ samples (i.e., *KIT*, *CTNNB1*, *MET*, *KRAS*, *HRAS*, *SMAD4*, *PDGFRA*, and *FGFR2*) or in *BRAF*^V600^ samples (i.e., *MAP2K1*, *PIK3CA*, *STK11*, *GNAS*, and *GNA11*). Finally, we compared the percentage of mutated genes in *BRAF*^WT^ and *BRAF*^V600^ samples according to the presence of one or several of co-occurring genetic alterations (Figure 2C).

### 2.3. Correlation between Clinicopathological Features with BRAF^V600^ Mutation and TERT Promoter Mutation

Then, we tested whether *BRAF* and *TERT* promoter mutations were correlated with the clinicopathological features in the cohort analyzed by NGS (Table 1). *BRAF*^V600^ melanomas significantly occurred in younger patients, with a median age of 57 years old versus 73 years, (*p* < 0.001) (Figure S1). Regarding primary tumor sites, their locations differed between the two groups, with 21 (39.6%) *BRAF*^V600^ primary tumors located on trunk and 18 (53%) *BRAF*^WT^ on limbs, including 5 (14.7%) acral melanomas (*p* = 0.06). Clark level also tends to be lower in *BRAF*^V600^ samples (*p* = 0.09). Conversely, no statistical differences were found for histologic subtypes, Breslow thickness, AJCC (American Joint Committee on Cancer) stage at the diagnosis, and patients’ sex between *BRAF*^V600^ and *BRAF*^WT^ melanomas. For *TERT* promoter mutational status, a significant association was observed with histological subtypes (*p* = 0.03) and with primary tumor localization (*p =* 0.003), but not with age, sex, Breslow thickness, Clark level, nor AJCC stage.

### 2.4. Prognostic Factors and PFS in BRAF^V600^ Samples

The prognostic value of clinical parameters and mutational status was next assessed for PFS under targeted therapy in *BRAF*^V600^ samples (Table 2, Table S2). All the *BRAF*^V600^ patients were treated with MAPK inhibitors: 6 received a monotherapy of BRAF inhibitor (vemurafenib (*n* = 4) or dabrafenib (*n* = 2)), 46 received a bitherapy of BRAF and MEK inhibitor (vemurafenib-cobimetinib (*n* = 4) or dabrafenib-trametinib (*n* = 42)), and one patient was treated alternately by bitherapy and monotherapy owing to poor tolerance. There were no differences in terms of PFS between the patients treated by monotherapy or bitherapy (*p* = 0.95). As expected, an elevated level of LDH in patient plasma was significantly associated with a shorter PFS (*p* = 0.01), while the presence of brain metastasis was near significant (*p* = 0.09). Regarding the mutational status of the samples, no significant differences were observed in terms of PFS for patients with or without co-occurring aberrations (*p* = 0.12), or in presence or absence of mutation on *TERT* promoter, *CDKN2A*, or *PTEN*, the most commonly altered genes in our cohort (*p* = 0.13, 0.34, and 0.35, respectively, Table 2).

**Figure 3:**
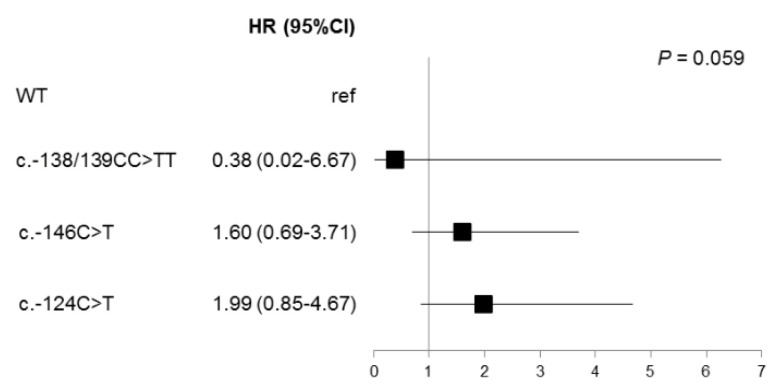
Univariate analysis of *TERT* promoter mutations with regard to PFS in *BRAF*^V600^ samples. Forest plot showing the hazard ratio for PFS associated to *TERT* promoter mutational status. *TERT* wild type (WT) samples were taken as reference (ref).

However, when *TERT* mutations were considered separately, a trend was observed (*p* = 0.059) with a longer PFS for patients harboring a c.-124C>T mutation (Figure 3). When these parameters were entered in a multivariate Cox model, the prognostic significance of the LDH level, brain metastasis, and *TERT* c.-124C>T mutational status remained in the model (Table 3), demonstrating that these biomarkers are independent prognostic markers of PFS.

**Figure 4:**
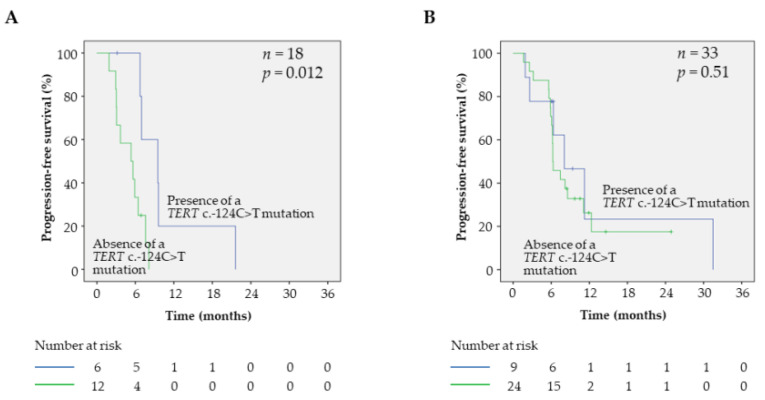
Effect of *TERT* c.-124C>T mutation on clinical outcome of *BRAF*^V600^ patients with or without brain metastasis. (**A**) Kaplan–Meier analyses of PFS in patients with brain metastasis in function of the *TERT* c.-124C>T mutation status. (**B**) Kaplan–Meier analyses of PFS in patients without brain metastasis in function of the *TERT* c.-124C>T mutation status.

To better evaluate the added prognostic value of the *TERT* c.-124C>T mutation, we further assessed it in subgroups of patients with different predicted outcomes. Interestingly, the presence of a *TERT* c.-124C>T mutation had a significant prognostic value in patients with brain metastasis (*p* = 0.01, Figure 4A), but not in patients without (*p* = 0.51, Figure 4B). The same tendency was also observed in patients with an elevated level of LDH in serum (*p* = 0.057) compared with patients with a normal LDH level (*p* = 0.403, Figure S2), suggesting that the presence of a *TERT* c.-124C>T mutation can represent a marker of good prognosis in subgroups of patients with poor prognosis (presence of brain metastasis and elevated level of LDH).

**Figure 5:**
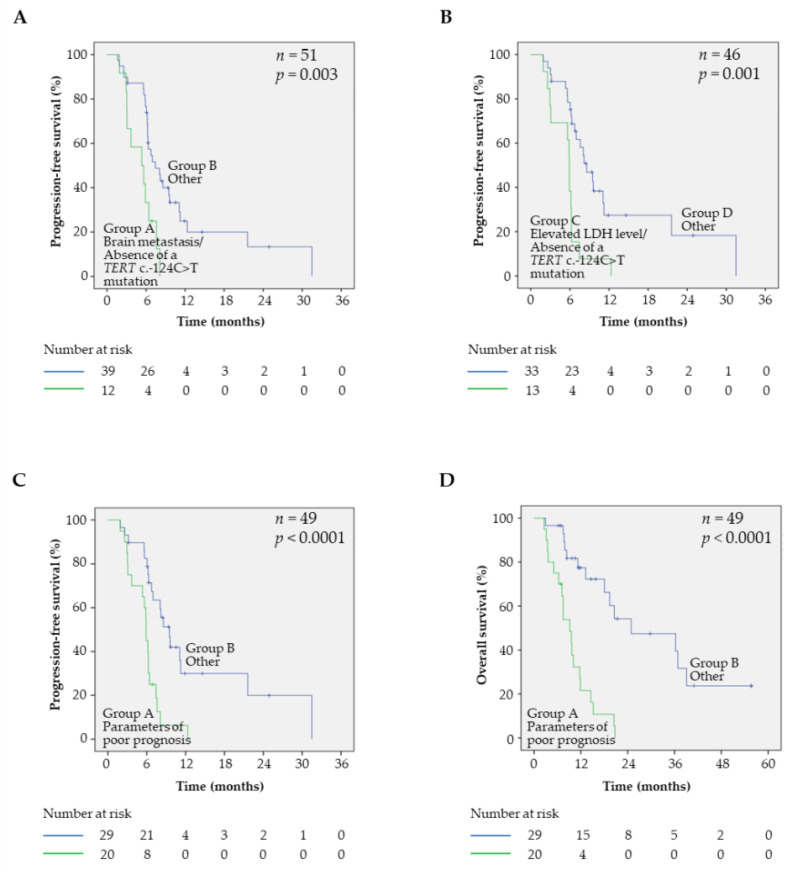
Prognostic value of a signature combining parameters of poor prognosis: elevated LDH level, brain metastasis, and absence of *TERT* c.-124C>T promoter mutation. (**A**) Kaplan–Meier analyses of PFS in patients combining brain metastasis and absence of *TERT* c.-124C>T promoter mutation (group A, green curve) and in the remaining patients (group B, blue curve). (**B**) Kaplan–Meier analyses of PFS in patients combining elevated LDH level and absence of the *TERT* c.-124C>T promoter mutation (group A, green curve) and in the remaining patients (group B, blue curve). (**C**) Kaplan–Meier analyses of PFS in patients combining parameters of poor prognosis (group A, green curve) and in the remaining patients (group B, blue curve). (**D**) Kaplan–Meier analyses of overall survival (OS) in patients combining parameters of poor prognosis (group A, green curve) and in the remaining patients (group B, blue curve).

These observations prompt us to evaluate signatures that combine the *TERT* mutation status with other prognostic markers. Thus, patients with brain metastasis in absence of *TERT* c.-124C>T mutation had a worse outcome than other patients (*p* = 0.003, Figure 5A). This signature was identified as the best fit for predicting worse clinical outcomes (likelihood = 243.55), relative to the model with the brain metastasis status alone or the *TERT* mutation status alone (likelihood = 248.01 with *p* = 0.034 and likelihood = 248.81 with *p* = 0.022, respectively). The same trend was observed for the LDH level parameter, as patients with an elevated LDH level in the absence of *TERT* c.-124C>T mutation had a worse outcome than others (*p* = 0.001, Figure 5B), and the signature combining the *TERT* c.-124C>T mutation status and LDH level had a better fit for predicting worse clinical outcomes (likelihood = 203.45) than models with the LDH level alone or the *TERT* mutation status alone (likelihood = 206.34 with *p* = 0.08 and likelihood = 210.95 with *p* = 0.006, respectively). Finally, the combination of parameters having poor prognosis (e.g., elevated LDH level and/or presence of brain metastasis and absence of *TERT* c.-124C>T mutation) had a worse outcome in terms of PFS than other patients (*p* < 0.0001, Figure 5C). Finally, this signature was the best fit for identifying patients of poor prognosis (likelihood = 201.78) compared with LDH level alone (likelihood = 206.34, *p* = 0.033), brain metastasis alone (likelihood = 209.81, *p* = 0.005), or *TERT* mutation status alone (likelihood = 210.96, *p* = 0.002).

### 2.5. Prognostic Factors and OS in BRAF^V600^ Samples

The prognostic value of clinical parameters and mutational status was also evaluated for OS in *BRAF*^V600^ samples (Table 4). We observed that sex and presence of brain metastasis were significantly correlated with OS. As for PFS, OS was not differentially affected by the presence or absence of co-occurring genetic alterations in the samples (*p* = 0.39), or *TERT* promoter, *CDKN2A*, or *PTEN* mutational status (*p* = 0.15, 0.45, and 0.39, respectively, Table 4), whereas there was a statistically significant difference between the *TERT* promoter mutation types (*p* = 0.002) (Figure S3). Thus, the specific effect of the *TERT* c.-124C>T mutation was retrieved, with longer OS in the subsets of patients with elevated LDH level (*p* = 0.006) or brain metastasis (*p* = 0.04), as well as in patients with normal LDH level (*p* = 0.05) and without brain metastasis (*p* = 0.005) (Figure S4). As observed for PFS, patients displaying a combination of parameters of poor prognosis (i.e., elevated level of LDH and/or presence of brain metastasis and absence of *TERT* c.-124C>T mutation) had a worse outcome in terms of OS than other patients (*p* < 0.0001, Figure 5D).

## 3. Discussion

Important steps have been taken over the past decade to better characterize the somatic melanoma mutation landscape, thanks to numerous high-throughput sequencing initiatives such as whole genome or whole exome sequencing [8,12,14,15,16]. Thus, the cutaneous melanoma genome has a very high and UV-specific mutational burden. Despite these great advances, the segregation between driver and passenger mutations, the cooperation of mutations, as well as the precise contribution in oncogenesis of each alteration in a same tumor remain elusive. To investigate these crucial considerations, we assessed the mutational frequency of well-known targetable-associated genes in a cohort of 89 cutaneous melanoma samples. We identified high mutational rates within *BRAF, NRAS*, and *TERT* promoter, as well as in *CDKN2A*, *PTEN, ERBB4,* and *RAC1*. Among them, mutation of the *TERT* promoter appeared as a frequent co-occurring mutation in both *BRAF*^V600^ and *BRAF*^WT^ patients. Of the utmost interest, we observed that specific *TERT* promoter mutation c.-124C>T displayed a statistically significant correlation with MAPK inhibitor treatment efficacy, specifically in a subset of *BRAF*^V600^ melanoma patients that had poorer PFS and OS. Importantly, this independent prognosis feature remains true for overall survival.

We observed a *BRAF*^V600^ mutation in 53 out of 89 (59.6%) melanomas, with specific *BRAF*^V600E^ and *BRAF*^V600K^ mutations in 45 and 8 patients, respectively. When we assessed the associations between *BRAF* genotyping with clinicopathological features, we found that *BRAF*^V600^ mutation was correlated with patient age (*p* < 0.001) and primary tumor location (*p =* 0.06), as previously reported [17,18]. In our cohort, no correlation was observed between *BRAF*^V600^ mutation status and increasing thickness (*p* = 0.23) or AJCC stage at the diagnosis (*p* = 0.56). Apart from its theragnostic implications, the prognosis value of *BRAF*^V600^ in melanoma remain controversial; several studies reported association of *BRAF* mutations with reduced survival [17,19,20], whereas others did not [18,21,22]. This suggests that *BRAF*^V600^ mutation alone is an insufficient predictive marker of biological aggressiveness.

We then compared the genomic profiling in *BRAF*^V600^ and *BRAF*^WT^ patients and found that *NRAS* was differentially mutated between the two groups (*p* < 0.001). This was expected in the context of the molecular landscape of melanomas that can be divided into four distinct subclasses, including BRAF subtype and NRAS subtype [23]. However, *NRAS* and *BRAF* mutation did co-occur in two samples. These two samples belonged to patients with recurrent metastatic melanomas that have been previously treated with MAPK inhibitors, which is consistent with the notion that *NRAS* mutation can arise through a resistance mechanism following targeted therapy [24]. Among the three remaining samples obtained after targeted therapy, one presented a mutation known to induce secondary resistance to targeted therapy (i.e., a deletion-insertion of *MAP2K1*), whereas the two others did not. Of note, *PTEN* and *RAC1* mutations—leading to the activation of PI3K/AKT pathway and to a MAPK inhibitor primary resistance [14,16]—were also observed in eight and two *BRAF*^V600^ samples, respectively.

No significant differences in terms of PFS were found in the presence or absence of co-occurring aberrations in *BRAF*^V600^ samples (*p* = 0.12). This can probably be explained by the lack of statistical power owing to the limited number of samples harboring a single *BRAF* mutation (*n* = 7, 13.2% of the *BRAF*^V600^ samples). However, this subject would need further explorations because the prognostic value of such co-existing genetic alterations has been demonstrated in melanoma [9,25] and in other cancers (e.g., in non-small cell lung carcinoma harboring activating *EGFR* mutations [26,27]).

*TERT* promoter mutations are currently detected in ~40% of melanomas, with mutation frequencies varying between melanoma subtypes. These mutations are most frequent in melanomas arising in non-acral skin and less frequent in mucosal and acral melanomas. Here, *TERT* promoter alterations were the most frequent genomic alteration identified. As our cohort was mainly composed of non-acral cutaneous melanomas, *TERT* promoter mutations occurred in 61 (68.5%) samples, which fitted well with previous reports [27,28,29].

*BRAF*^V600^ have been reported to cooperate with *TERT* promoter mutations more frequently than with any other mutations, upregulating *TERT* mutant expression via the FOS/GABP pathway [28,30,31,32,33,34,35]. Thus, Vinagre et al. [36] demonstrated the association between *BRAF* and *TERT* promoter mutations in melanoma, and that *TERT* messenger RNA (mRNA) levels are higher when *TERT* promoter and *BRAF* mutations coexist in thyroid cancers. In our cohort, the frequency of *TERT* promoter alterations tended to be higher in *BRAF*^V600^ samples, with 39 mutations in 53 samples (73.6%), than in *BRAF*^WT^ samples, with 22 mutations in 36 samples (61.1%), although this difference did not reach statistical significance. We did not observe any correlations between *TERT* promoter mutation rates and age, sex, and AJCC stage at the time of diagnosis. Other authors did report significant correlations between *TERT* promoter mutations, age, and regional and distant metastases [31,37]. This discrepancy may be owing to the small number of cases in our study.

*TERT* encodes the reverse transcriptase component of the telomerase complex, which is necessary for chromosomal telomeres length stabilization and promotion of cell survival. c.-124C>T and c.-146C>T *TERT* promoter mutations have been described to create de novo E twenty-six (ETS) binding motifs, inducing upregulation of *TERT* mRNA and increased telomerase activity in malignant cells. However, TERT expression seems to be differentially enhanced by *TERT* promoter mutations, with a greater effect of c.-124C>T mutation [38]. This mechanism could be modulated by the presence or absence of the *rs2853669* polymorphism at the −245 bp position, which is described to disrupt a pre-existing ETS2 binding site [39]. *TERT* promoter mutations have been collectively associated with more aggressive melanomas and poorer outcomes, allowing to propose *TERT* promoter as a poor prognostic factor [28,32]. However, with the exception of the c.-138/-139CC>TT mutation, which is associated with the worst survival in several studies [40,41], the specific effect of each mutation is still debated. Discordant results have been published regarding the c.-146C>T mutation, with a better disease-free survival in comparison with other *TERT* mutants in one study [41], and a poorer PFS in others [34,40]. In the very recent study of Del Bianco et al. [34], median PFS of patients with c.-124C>T mutation was found to be 9.5 months, versus 5.4 months for patients with c.-146C>T mutation. In our study, the c.-124C>T mutation was associated with better survival compared with the c.-146C>T mutation, but only in poor prognosis patients harboring brain metastasis or elevated serum LDH levels, confirming the different role of both *TERT* mutations on the MAPK pathway blockade. Interestingly, in the multivariate analysis, c.-124C>T mutation remained significantly associated with brain metastasis (*n* = 51, *p* = 0.04) and elevated LDH (*n* = 46, *p* = 0.006). The c.-138/-139CC>TT *TERT* promoter mutation was present in 2 *BRAF*^V600^ samples, which was too low to confirm its worse prognostic value. Nevertheless, one of the patients who harbored this mutation was indeed a low responder, with a PFS of 1.8 months, while the second had a PFS of 6.0 months, which was close to the median PFS of *BRAF*^V600^ samples (6.1 months). *TERT* promoter mutations may also have a different impact on patient outcome according to the presence or not of the *rs2853669* polymorphism. Indeed, the effect of *TERT* promoter mutations on survival seems to be enhanced in melanoma patients that did not carry the polymorphism [33]. However, discordant results have been reported in glioblastoma [42,43,44,45]. As the corresponding position was not included in our panel, the link between *TERT* promoter mutations and this polymorphism and its effect on patient survival could not be unraveled in our study and would require further investigations.

Together, these data provided insights into the recurrent genomic aberrations associated with clinical relevance and demonstrated how these insights could inform triage of patients for effective precision cancer treatments. We observed that MAPK inhibitors treatment efficacy and patients’ PFS, in the presence of elevated LDH level and brain metastasis, depended on the mutation type of the *TERT* promoter, highlighting, first, the functional link that exists between TERT biology and the MAPK pathway, and second, a specific behavior of the *TERT* c.-124C>T mutations in a specific subset of poor prognosis melanomas. Finally, there is no direct evidence that *TERT* promoter aberrations have a key role in melanoma progression, but this genotyping needs to be confirmed in future research. The challenging, but essential task now is to seek confirmation in an independent and prospective collection of additional datasets. If validated in larger cohorts, *TERT* promoter mutations may be used in clinical practice for its therapeutic relevance, either in terms of influencing the efficacy of established therapies (e.g., MAPK inhibitors or immunotherapies) or they might even prove to be valuable direct therapeutic targets.

## 4. Materials and Methods

### 4.1. Patient and Sample Collection

From 2014 to 2019, 113 patients with a cutaneous melanoma underwent NGS analysis at the University Hospital of Montpellier. All primary samples were obtained prior to any treatment initiation, as well as most of the metastasis samples, with the exception of five recurrent metastatic *BRAF*^V600^ melanomas that have been previously treated by targeted therapy. The noninterventional study was conducted in accordance with local ethical guidelines and was reviewed by the Ethical Committee from the Montpellier University Hospital (March 2019). Samples were obtained following research project approval by the Institutional Review Board from CRB-CHUM (BB-0033-00031). An approved informed consent statement was acquired for all patients. All corresponding lesions were excised and submitted for standard pathological examination. The percentage of tumor cells in the series ranged from 30 to 100% (Table S2). Tissue punches using a 1 mm needle or macrodissected 10 μm thick section were performed from tumor paraffin blocks to increase the percentage of tumor cells in the sample. Genomic DNA was extracted using the Maxwell^®^ RSC DNA FFPE Kit (Promega, Madison, WI, USA) according to the manufacturer’s recommendations. Twenty-four patients were excluded from the study owing to lost-to-follow-up, absence of targeted therapy for *BRAF*^V600^ patients, and/or poor quality of extracted DNA (Figure 1). Medical records were reviewed to extract clinicopathological data, including sex, age, diagnoses, therapeutic agents, and survival.

### 4.2. NGS Analysis

Library preparation was performed as previously described [46]. Briefly, extracted DNA was qualified using KAPA Sybr^®^ Fast qPCR (Kapa Biosystems, Boston, MA, USA). A home-made panel targeting specific exons of 35 clinically relevant cancer genes was used for amplification of regions of interest (Table S3). For each sample, dual-strand libraries were prepared using a TruSeq Custom Amplicon protocol, as described by the manufacturer (Illumina, Evry, France). After amplification, PCR products were purified using AMPure XP beads (Beckman Coulter, Brea, CA, USA), quantified, normalized, and pair-end sequenced on a MiSeq instrument (2 × 150 cycles, Illumina). After sequencing, the four FastQ files generated per samples were automatically analysed using a bioinformatic workflow managed by Jflow [47]. Briefly, reads were trimmed with cutadapt (version 1.18) [48] and aligned to the human genome GRCh37 with BWA (version 0.7.17) [49], and the variant calling was performed using VarDict (version 1.6.0). Variants present in both libraries with a variant allele frequency >5% and a depth coverage of 300× or greater were then annotated with Variant Effect Predictor (version 94) [50] and reported. Variants having a frequency of 1% or more in the population (in database Exome Aggregation Consortium (ExAC) Variants, Variants Exome Sequencing Project (ESP), or 1000 Genomes Project) were considered as polymorphisms and were excluded.

### 4.3. Statistical Analysis

The relationship between the patients’ clinicopathological characteristics and other markers was analyzed using the χ^2^ test or the Fisher test depending on the number of patients per group. Mann–Whitney analysis was performed using Statgraphics (Statgraphics Centurion, Neuilly sur Seine, France). The significance of differences between survival rates was estimated using the Kaplan–Meier method and ascertained with the log-rank test using SPSS^®^ Software (SPSS Inc., Armonk, NY, USA). When the number of samples analyzed was low (*n* < 20), a bootstrap based log-rank method using 1000 bootstrap constructed samples was used to ascertain the statistical significance of the results. Candidate prognostic factors for PFS with a 0.1 significance level in univariate analysis were entered in a multivariate Cox model, and a backward selection procedure was used to determine independent prognostic markers. A likelihood ratio test was applied to select the best fit between models. A *p*-value < 0.05 was considered significant.

## 5. Conclusions

In our study, *TERT* c.-124C>T mutation was predictive of a better clinical outcome in a subset of poor prognosis *BRAF*^V600^ patients under MAPK inhibitors. Altogether, given that adaptive therapeutic strategies are required to overcome acquired resistance in *BRAF*^V600^ melanoma patients, we believe that our findings will have relevant clinical implications for patient management and that *TERT* promoter mutation detection should be considered to better anticipate patient relapse and introduction of immunotherapy. This first line of evidence calls for further explorations in larger cohorts and should be considered in the design and interpretation of future clinical trials.

## Figures and Tables

**Table 1:** Correlation between clinicopathological features and *BRAF* and *TERT* promoter mutational status.

Clinicopathological Features		*BRAF* Status			*TERT* Promoter Status		
		*BRAF*^WT^ (*n* = 36)	*BRAF*^V600^ (*n* = 53)	*p*-Value	WT (*n* = 28)	Mutated (*n* = 61)	*p*-Value
Age	≤60	4 (11.1)	28 (52.8)	<0.001	9 (32.1)	23 (37.7)	0.61
	>60	32 (88.9)	25 (47.2)		19 (67.9)	38 (62.3)	
Sex	Male	22 (61.1)	28 (52.8)	0.44	15 (53.6)	35 (57.4)	0.74
	Female	14 (38.9)	25 (47.2)		13 (46.4)	26 (42.6)	
Histological type	NM	8 (25.8)	10 (20.8)	0.18	5 (20)	13 (24.1)	0.03
	SSM	15 (48.4)	24 (50)		10 (40)	29 (53.7)	
	MUP	3 (9.7)	9 (18.8)		3 (12)	9 (16.7)	
	Unclassified	0 (0)	3 (6.3)		1 (4)	2 (3.7)	
	ALM	5 (16.1)	2 (4.2)		6 (24)	1 (1.9)	
	Missing data	5	5		3	7	
Primary tumor site	Head/neck	7 (20.6)	11 (20.8)	0.06	1 (3.7)	17 (28.3)	0.003
	Upper limbs	4 (11.8)	2 (3.8)		1 (3.7)	5 (8.3)	
	Trunk	6 (17.6)	21 (39.6)		8 (29.6)	19 (31.7)	
	Lower limbs	9 (26.5)	8 (15.1)		8 (29.6)	9 (15.0)	
	MUP	3 (8.8)	9 (17)		3 (11.1)	9 (15.0)	
	Acral	5 (14.7)	2 (3.8)		6 (22.2)	1 (1.7)	
	Missing data	2	0		1	1	
Breslow thickness *	<1 mm	1 (3.3)	4 (10.2)	0.25	2 (8.7)	3 (6.5)	0.66
	1–1.99 mm	4 (13.3)	11 (28.2)		3 (13.0)	12 (26.1)	
	2–3.99 mm	8 (26.6)	9 (23.1)		6 (26.1)	11 (23.9)	
	≥4 mm	17 (56.6)	15 (38.4)		12 (52.2)	20 (43.5)	
	Missing data	3	5		2	6	
Clark level *	II	0 (0)	2 (5.4)	0.09	0 (0)	2 (4.6)	0.66
	III	2 (6.9)	10 (27.0)		3 (13.6)	9 (20.4)	
	IV	21 (72.4)	20 (54.1)		15 (68.2)	26 (59.1)	
	V	6 (20.7)	5 (13.5)		4 (18.2)	7 (15.9)	
	Missing data	4	7		3	8	
AJCC	I	3 (8.8)	7 (14.0)	0.56	3 (11.1)	7 (12.3)	0.97
	II	16 (47.1)	17 (34.0)		10 (37.0)	23 (40.4)	
	III	8 (23.5)	11 (22.0)		6 (22.2)	13 (22.8)	
	IV	7 (20.6)	15 (30.0)		8 (29.6)	14 (24.6)	
	Missing data	2	3		1	4	

WT: wild-type, NM: nodular melanoma, SSM: superficial spreading melanoma, MUP: melanoma of unknown primary, ALM: acral lentiginous melanoma, AJCC: American Joint Committee on Cancer. * MUP are not taken into account for Breslow thickness and Clark level.

**Table 2:** Univariate analysis of clinical parameters and sample mutational status with regard to progression-free survival (PFS) in *BRAF*^V600^ patients.

Clinical Parameters	Samples (*n*)	HR	95% CI	*p*-Value
Sex (Male; Female)	51	1.16	0.61–2.20	NS (0.65)
Age (≤60; >60-year-old)	51	1.66	0.88–3.14	NS (0.12)
LDH level (normal; high)	46	2.38	1.19–4.75	0.01
Brain metastases (absence; presence)	51	1.72	0.90–3.29	NS (0.09)
Histological type (NM; SSM; MUP; ALM; unclassified)	45	1.35	0.93–1.95	NS (0.11)
Breslow thickness (<1 mm; 1–2 mm; 2–4 mm; >4)	37	1.10	0.75–1.62	NS (0.61)
Treatment modalities (monotherapy; bitherapy)	50	1.03	0.40–2.65	NS (0.95)
Co-occurring mutation (absence; presence)	51	0.49	0.20–1.20	NS (0.12)
*TERT* promoter mutation (absence; presence)	51	0.58	0.29–1.17	NS (0.13)
*CDKN2A* mutation (absence; presence)	51	1.50	0.65–3.44	NS (0.34)
*PTEN* mutation (absence; presence)	51	1.78	0.65–3.40	NS (0.35)

HR: hazard ratio, 95% CI: 95% confidence interval, NM: nodular melanoma, SSM: superficial spreading melanoma, MUP: melanoma of unknown primary, ALM: acral lentiginous melanoma, LDH: lactate dehydrogenase, NS: not significant.

**Table 3:** Multivariate analysis of clinical parameters and *TERT* c.-124C>T mutational status with regard to PFS in *BRAF*^V600^ patients.

Clinical Parameters	HR	95% CI	*p*-Value
LDH level (normal; high)	2.86	1.36–6.02	0.006
Brain metastasis (absence; presence)	2.34	1.03–5.30	0.04
*TERT* c.-124C>T mutation (absence; presence)	1.37	1.08–1.73	0.009

HR: hazard ratio, 95%CI: 95% confidence interval, LDH: lactate dehydrogenase.

**Table 4:** Univariate analysis of clinical parameters and sample mutational status with regard to overall survival (OS) in *BRAF*^V600^ patients.

Clinical Parameters	Samples (n)	HR	95% CI	*p*-Value
Sex (Male; Female)	51	2.20	1.10–4.42	0.03
Age (≤60; >60-year-old)	51	1.63	0.83–3.20	NS (0.15)
LDH level (normal; high)	46	1.71	0.83–3.52	NS (0.15)
Brain metastases (absence; presence)	51	2.21	1.08–4.50	0.03
Histological type (NM; SSM; MUP; ALM; unclassified)	45	1.27	0.89–1.82	NS (0.19)
Breslow thickness (<1 mm; 1–2 mm; 2–4 mm; >4)	37	1.02	0.67–1.55	NS (0.93)
Treatment modalities (monotherapy; bitherapy)	50	0.61	0.25–1.49	NS (0.27)
Co-occurring mutation (absence; presence)	51	0.68	0.28–1.65	NS (0.39)
*TERT* promoter mutation (absence; presence)	51	0.58	0.28–1.21	NS (0.15)
*CDKN2A* mutation (absence; presence)	51	1.41	0.58–3.45	NS (0.45)
*PTEN* mutation (absence; presence)	51	1.44	0.63–3.31	NS (0.39)

HR: hazard ratio, 95% CI: 95% confidence interval, NM: nodular melanoma, SSM: superficial spreading melanoma, MUP: melanoma of unknown primary, ALM: acral lentiginous melanoma.

## Supplementary Materials

The following are available online at https://www.mdpi.com/2072-6694/12/8/2224/s1, Figure S1: Age repartition in *BRAF*^V600^ and *BRAF*^WT^ samples, Figure S2: Effect of *TERT* c.-124C>T promoter mutation on clinical outcome of *BRAF*^V600^ patients with elevated or normal LDH level, Figure S3: Univariate analysis of *TERT* promoter mutations with regard to OS in *BRAF*^V600^ samples, Figure S4: Effect of *TERT* c.-124C>T mutation on OS of *BRAF*^V600^ patients, Table S1: Cohort clinicopathological features, Table S2: Clinical prognostic factors and targeted therapy modalities in *BRAF*^V600^ samples, Table S3: Sample characteristics, Table S4: NGS panel.
