# Supplementary material for: TERT Promoter Mutation as an Independent Prognostic Marker for Poor Prognosis MAPK Inhibitors-Treated Melanoma
Source: Cancers (Basel). 2020 Aug 9;12(8):2224. doi: 10.3390/cancers12082224 (PMC7463448; doi:10.3390/cancers12082224)
Supplement: Supplementary file 1 [file cancers-12-02224-s001.pdf]

cancers

Article

TERT Promoter Mutation as an Independent Prognostic Marker for Poor Prognosis MAPK Inhibitors-Treated Melanoma

Pauline Blateau, Etienne Coyaud, Estelle Laurent, Benoit Béganton, Vincent Ducros, Géraldine Chauchard, Julie A. Vendrell and Jérôme Solassol

Supplementary Materials

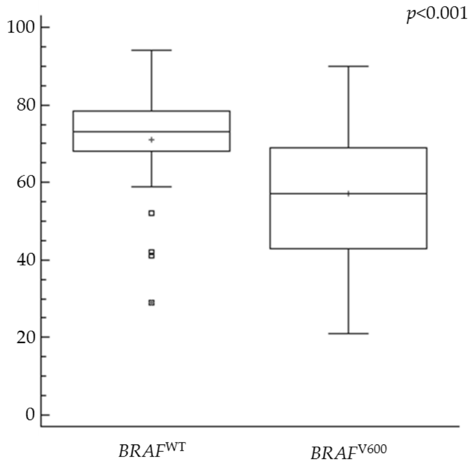

Figure S1. Age repartition in *BRAF*^{V600} and *BRAF*^{WT} samples.

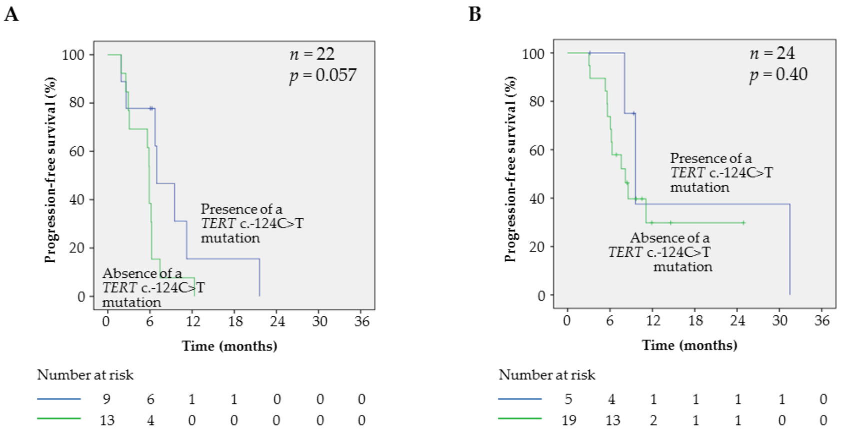

Figure S2. Effect of *TERT* c.-124C>T promoter mutation on clinical outcome of *BRAF*^{V600} patients with elevated or normal LDH level. (**A**) Kaplan-Meier analysis of PFS in patients with elevated LDH level in function of the *TERT* c.-124C>T promoter mutation status. (**B**) Kaplan-Meier analysis of PFS in patients with normal LDH level in function of the *TERT* c.-124C>T promoter mutation status.

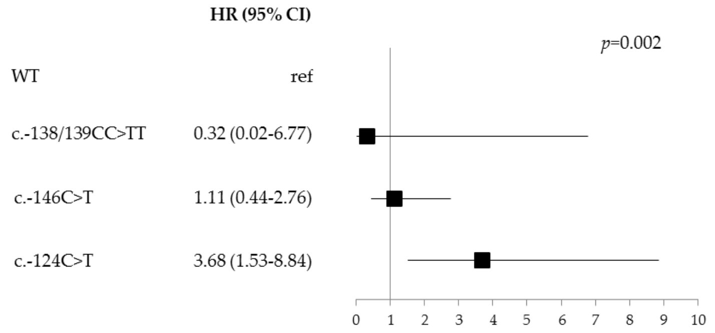

Figure S3. Univariate analysis of *TERT* promoter mutations with regard to OS in *BRAF*^{V600} samples**.** Forest plot showing the hazard ratio for PFS associated to *TERT* promoter mutational status. *TERT* wild type (WT) samples were taken as reference (ref).

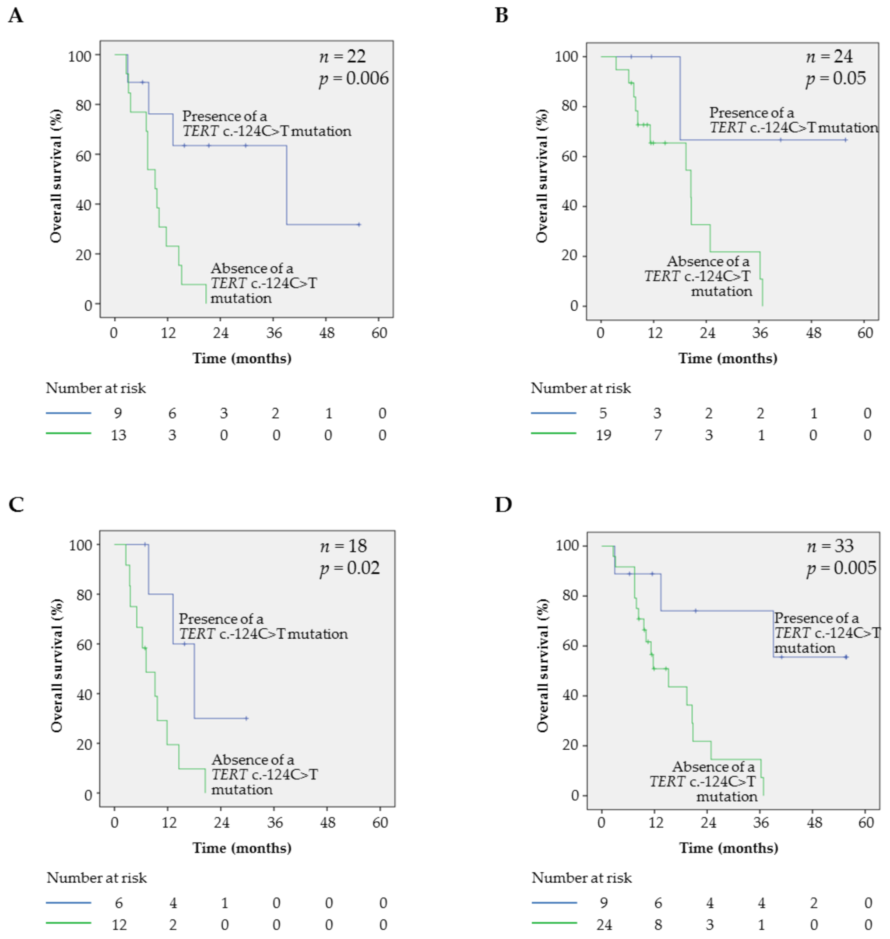

Figure S4. Effect of *TERT* c.-124C>T mutation on OS of *BRAF*^{V600} patients depending on LDH level and brain metastasis presence. (**A**) Kaplan-Meier analysis of OS in patients with elevated LDH level in function of the *TERT* c.-124C>T promoter mutation status. (**B**) Kaplan-Meier analysis of OS in patients with normal LDH level in function of the *TERT* c.-124C>T promoter mutation status. (**C**) Kaplan-Meier analysis of OS in patients with brain metastasis in function of the *TERT* c.-124C>T promoter mutation status. (**D**) Kaplan-Meier analysis of OS in patients without brain metastasis in function of the *TERT* c.-124C>T promoter mutation status.

Table S1. Cohort clinicopathological features.

Clinicopathological features		*n* (%)
Age	<60	32 (36.0)
	>60	57 (64.0)
Sex	Male	50 (56.2)
	Female	39 (43.8)
Histological type	NM	18 (22.8)
	SSM	39 (49.4)
	MUP	12 (15.2)
	Unclassified	3 (3.8)
	ALM	7 (8.9)
	Missing data	10
Primary tumor site	Head/neck	18 (20.7)
	Upper limbs	6 (6.9)
	Trunk	27 (31.0)
	Lower limbs	17 (19.6)
	MUP	12 (13.8)
	Acral	7 (8.0)
	Missing data	2
Breslow thickness*	<1 mm	5 (7.2)
	1–1.99 mm	15 (21.7)
	2–3.99 mm	17 (24.6)
	≥4 mm	32 (46.4)
	Missing data	8
Clark level*	II	2 (3.0)
	III	12 (18.2)
	IV	41 (62.1)
	V	11 (16.7)
	Missing data	11
AJCC	I	10 (11.9)
	II	33 (39.3)
	III	19 (22.6)
	IV	22 (26.2)
	Missing data	5

Table S2. Clinical prognostic factors and targeted therapy modalities in *BRAF*^{V600} samples.

**Clinical Prognostic Factors**	***BRAF*^{V600}**	**(*n* = 53) (%)**
AJCC stage at the initiation of MAPK inhibitor	III	6 (11.3)
	IV	47 (88.7)
Number of metastasis	0	8 (15.1)
	1	2 (3.8)
	2	2 (3.8)
	3	6 (11.3)
	4	2 (3.8)
	≥5	33 (62.3)
Brain metastasis	Absence	35 (66.0)
	Presence	18 (34.0)
LDH	Normal	25 (52.1)
	Elevated	23 (47.9)
Targeted therapy modalities		
BRAFi monotherapy	Vemurafenib	4 (7.7)
	Dabrafenib	2 (3.8)
BRAFi and MEKi bitherapy	Vemurafenib–Cobimetinib	4 (7.7)
	Dabrafenib–Trametinib	42 (80.8)

AJCC: American Joint Committee on Cancer, MAPK: Mitogen-Activated Protein Kinases.

Table S3. Samples characteristics.

Tumoral Status	*n* (%)	Median Tumor Content (Min-Max)	Samples Obtained Pre-treatment *n* (%)
All	89 (100)	80% (30–100)	84 (94.4)
Primary tumor	36 (40.4)	75% (30–90)	36 (100.0)
Metastasis	53 (59.6)	80% (30–100)	48 (90.6)

Table S4. NGS panel.

Gene	Exons	Reference Transcripts
AKT1	3	(NM_001014431)
ALK	20, 21, 22, 23, 24, 25	(NM_004304)
BRAF	11, 15	(NM_004333)
CDKN2A	1, 2, 3	(NM_000077)
CTNNB1	3	(NM_001904)
DDR2	17	(NM_006182)
EGFR	18, 19, 20, 21	(NM_005228)
ERBB2	20	(NM_004448)
ERBB4	10, 12	(NM_005235)
FGFR1	12, 14	(NM_023110)
FGFR2	7, 12, 14	(NM_000141)
FGFR3	7, 9, 14	(NM_000142)
GNA11	4, 5	(NM_002067)
GNAQ	5	(NM_002072)
GNAS	8, 9	(NM_000516)
H3F3A	2	(NM_002107)
H3F3B	2	(NM_005324)
HIST1H3B	1	(NM_003537)
HRAS	2, 3, 4	(NM_005343)
IDH1	4	(NM_005896)
IDH2	4	(NM_002168)
JAK2	12, 13, 14	(NM_004972)
KIT	8, 9, 11, 13, 17, 18	(NM_000222)
KRAS	2, 3, 4	(NM_033360)
MAP2K1	2	(NM_002755)
MET	2, 14, 15, 16, 17, 18, 19, 20	(NM_001127500)
NRAS	2, 3, 4	(NM_002524)
PDGFRA	12, 14, 18	(NM_006206)
PIK3CA	10, 21	(NM_006218)
POLE	9, 10, 11, 12, 13, 14	(NM_006231)
PTEN	1, 2, 3, 4, 5, 6, 7, 8, 9	(NM_000314)
RAC1	2	(NM_018890)
SMAD4	2, 3, 9, 10, 11, 12	(NM_005359)
STK11	1, 2, 3, 4, 5, 6, 7, 8, 9	(NM_000455)
TERT	Promoter	–
